# Anorexia Nervosa and Body Fat Distribution: A Systematic Review

**DOI:** 10.3390/nu6093895

**Published:** 2014-09-23

**Authors:** Marwan El Ghoch, Simona Calugi, Silvia Lamburghini, Riccardo Dalle Grave

**Affiliations:** Department of Eating and Weight Disorders, Villa Garda Hospital, Via Montebaldo, 89, 37016 Garda (Vr), Italy; E-Mails: si.calugi@gmail.com (S.C.); silvia.lambo@hotmail.it (S.L.); rdalleg@gmail.com (R.D.G.)

**Keywords:** anorexia nervosa, eating disorders, body composition, body fat percentage, body fat redistribution, adipose tissue distribution

## Abstract

The aim of this paper was to conduct a systematic review of body fat distribution before and after partial and complete weight restoration in individuals with anorexia nervosa. Literature searches, study selection, method development and quality appraisal were performed independently by two authors, and data was synthesized using a narrative approach. Twenty studies met the inclusion criteria and were consequently analyzed. The review had five main findings. First, during anorexia nervosa adolescent females lose more central body fat, while adult females more peripheral fat. Second, partial weight restoration leads to greater fat mass deposition in the trunk region than other body regions in adolescent females. Third, after short-term weight restoration, whether partial or complete, adults show a central adiposity phenotype with respect to healthy age-matched controls. Fourth, central fat distribution is associated with increased insulin resistance, but does not adversely affect eating disorder psychopathology or cause psychological distress in female adults. Fifth, the abnormal central fat distribution seems to normalize after long-term maintenance of complete weight restoration, indicating that preferential central distribution of body fat is a transitory phenomenon. However, a discrepancy in the findings has been noted, especially between adolescents and adults; besides age and gender, these appear to be related to differences in the methodology and time of body composition assessments. The PROSPERO Registry—Anorexia Nervosa and Body Fat Distribution: A Systematic Review (CRD42014008738).

## 1. Introduction

Anorexia nervosa is a health problem associated with physical and psychosocial morbidity, and increased risk of mortality [1]. Its cause is not yet completely clear [2] and as such it remains difficult to treat [3]. The core features of anorexia nervosa are restriction of energy intake relative to requirement, which leads to significantly low weight and body fat [4,5,6,7]. Hence the recovery of a normal body weight and body fat are key strategies in the treatment of anorexia nervosa [8,9,10,11,12], However, a common obstacle is the abdominal protrusion often observed by clinicians and patients during weight restoration [13] prompting the latter to resist further weight gain or to relapse [14]. In addition, the preferential accumulation of body fat after short-term weight restoration may have negative long-term metabolic consequences, as observed in other conditions (*i.e.*, obesity) [15]. Therefore, the study of the changes in body fat distribution during the process of weight restoration may have important clinical implications for treatment planners.

In recent years, a considerable body of research has been amassed to assess body fat and its distribution, both before and after partial and complete weight restoration in anorexia nervosa. In addition, the clinical significance of body fat changes after weight restoration on eating disorder psychopathology, psychological distress, and metabolic indices has recently come under focus, but no systematic review of this important clinical issue has yet been conducted.

We set out to systematically review the published literature to address the clinical issues regarding body fat distribution in adolescent and adult patients with anorexia nervosa before and after partial or complete weight restoration according to the PICO formulation [16], focusing in particular on its effect on eating disorder psychopathology, psychological distress, and metabolic indices. 

P-Population: individuals in the general population with anorexia nervosa. I-Intervention: partial or complete weight restoration. C-Comparison: anorexia nervosa group before and after partial or complete weight restoration (when available), and healthy control group (when available). O-Outcome: body fat distribution and its influence on eating disorder psychopathology, psychological distress, and metabolic indices.

## 2. Experimental Section

This report was completed in accordance with the Preferred Reporting Items for Systematic Review and Meta-Analyses (PRISMA) guidelines [17].

### 2.1. Inclusion and Exclusion Criteria

All studies that evaluated body fat distribution were taken into consideration. However, the review included only: (I) manuscripts in English; (II) original articles; (III) prospective or retrospective observational (analytical or descriptive), experimental or quasi-experimental studies. Non-original studies, including editorials and letters to the editor, were excluded.

### 2.2. Information Source and Search Strategy

Literature searches were initially performed on PubMed. The following MeSH terms, words, and combinations of words were used to perform the systematic search: #1 anorexia nervosa, #2 body composition, #3 body fat percentage, 4# body fat redistribution, #5 adipose tissue distribution, as were their intersections: (#1) AND (#2 OR #3 OR #4 OR #5). Additionally, reference lists of key studies were searched manually to retrieve any that had not been identified via the initial search strategy. No limitation on publication date was imposed.

### 2.3. Study Selection

Electronic literature searches and study selection on the basis of methodology and appropriateness for inclusion were performed independently and in duplicate by two authors (Marwan El Ghoch and Simona Calugi). The NICE guidelines checklist was used for quality appraisal in non-controlled longitudinal studies and cross-sectional studies [18]. A total score of 0–3 was considered poor quality; between 4–6, fair quality; and ≥7 good quality. Quality appraisal was conducted according to the Newcastle-Ottawa Scale (NOS) in the controlled longitudinal studies [19], wherein a 9-star system is employed for quality assessment. Scores of 4, 2 and 3 were respectively assigned to the criteria “selection of study groups”, “comparability of study groups”, “assessment of outcomes and adequacy of follow-up”. Studies with scores of 0–3, 4–6, and 7–9 were considered as low, moderate and high quality, respectively [20]. Disagreements between reviewers were resolved by consensus.

### 2.4. Data Collection Process and Data Items

As a first step, the title and abstract of each paper were screened for language and relevance of subject matter. In the next round, studies were assessed for methodological quality and appropriateness for inclusion. Details of the selected studies are presented in Table 1, which reports the authors, year of publication, type, sample, intervention and main findings of each study.

### 2.5. Data Synthesis

Studies deemed fit for inclusion were subjected to narrative synthesis. A narrative review is discursive in nature and seeks to summarize the current state of knowledge in relation to a particular domain by considering a wide variety of sources and reaching conclusions through reason or argument [21].

**Table 1:** Studies included in the systematic review.

First Author	Year	Method	Study	Sample	Intervention	BF Distribution before Weight Gain	BF Distribution after Partial/Complete Weight Restoration
**Adolescents**							
Kerruish *et al.* [22]	2002	DXA	Cross-sectional	23 adolescent AN females *vs.* 25 age-matched controls	-	Lower trunk fat, leg fat and trunk/leg fat ratio than in control subjects (less central fat)	-
Misra *et al.* [23]	2003	DXA	Longitudinal, over 1 year	21 adolescent AN females (only 13 had a BMI increase) *vs.* 21 age-matched controls	Complete weight restoration (BMI >10th percentile)	Lower percentage trunk fat than controls, whereas percentage extremity fat was not significantly different between the groups	Trunk fat and trunk/extremity fat ratio did not exceed that of controls, indicating that the changes in adolescent females with AN most likely represented a normalization of BF distribution.
Misra *et al.* [24]	2005	DXA	Longitudinal, over 1 year	23 adolescent AN females (only 11 obtained a BMI increase) *vs.* 20 age-matched controls	Complete weight restoration	Percentage trunk fat was significantly lower than in controls, whereas percentage extremity fat did not differ, suggesting preferential loss of trunk fat with weight loss	-
De Alvaro *et al.* [25]	2007	DXA	Longitudinal, over 24 months	42 adolescent AN-R females (only 15 achieved weight and menses recovery) *vs.* 23 controls	Slow complete weight restoration	Lower trunk/extremity fat ratio in prolonged malnutrition patients due to a greater loss of trunk fat.	Slow and complete weight restoration was associated with an adequately distributed fat mass acquisition, with no changes in regional fat percentages
Misra *et al.* [26]	2008	DXA	Cross-sectional	15 adolescent AN boys *vs.* 15 controls	-	Adolescent boys with AN had higher percentage trunk fat and trunk/extremity ratio than controls associated with lower testosterone concentrations	-
Franzoni *et al.* [27]	2014	DXA	Longitudinal, 1 year of treatment	46 adolescent AN-R females; no controls	Short-term partial weight restoration	Not mentioned	More evident deposition of fat in trunk region, in the absence of healthy control group
Forbes [28]	1990	AM	Cross-sectional	2 males and 30 females with AN (adolescents) aged 10–22 years *vs.* normative data	-	Regional adiposity was measured using waist and hip circumferences—these measures decreased, with no change in waist-to-hip ratio	-
**Adults**							
Kirchengast *et al.* [29]	1999	DXA	Cross-sectional	15 adult AN females *vs.* 15 age-matched controls	-	Underweight infertile AN patients showed hypergynoid distribution despite low oestrogen levels	-
Iketani *et al.* [30]	1999	DXA	Longitudinal, inpatient and outpatient treatments	21 adult AN females *vs.* 10 age-matched controls	Short-term partial weight restoration	BF reduction in all regions (trunk, pelvis, upper and lower extremities) but no mention of its relative distribution.	Trunk and pelvis BF increased remarkably and reached the levels of controls, but the upper and lower extremity BF remained below the control level.
Pagliato *et al.* [31]	2000	DXA	Longitudinal, inpatient treatment	17 adult AN patients; no controls	Short-term partial weight restoration	Not mentioned	In the inpatient group, the increase of BF in trunk region was higher with respect to other patterns
Grinspoon *et al.* [32]	2001	DXA	Longitudinal, outpatient treatment	27 adult AN females *vs.* 20 age-matched controls	Spontaneous partial weight restoration	Percentage trunk fat not statistically different between patients and controls, percentage extremity fat significantly lower than in controls, but trunk/extremity fat ratio no different to that in healthy controls	Increase in trunk adiposity. Estrogen administration did not appear to prevent such distribution
Kirchengast *et al.* [33]	2003	DXA	Cross-sectional	15 adult AN females *vs.* 19 age-matched controls	-	AN patients showed a gynoid fat pattern no different from healthy controls	-
Dellava *et al.* [34]	2010	DXA	Cross-sectional	16 adult AN females *vs.* 18 age- and BMI-matched controls	Long-term weight maintenance	-	Women recovered from AN for two years or more had similar body fat distribution to controls
Prioletta *et al.* [35]	2011	DXA	Longitudinal, 12-week multidisciplinary re-education program	19 AN females aged 17–32 years *vs.* 20 age-matched controls	Short-term partial weight restoration	No difference in percentage trunk fat with respect to controls	Minimum and short-term partial weight restoration led to a preferential redistribution of BF in trunk region with respect to controls, and such distribution was correlated with insulin-resistance status
El Ghoch *et al.* [36]	2014	DXA	Longitudinal, (20-week inpatient treatment)	50 adult AN females *vs.* 100 healthy, lean age- and BMI-matched controls	Short-term complete weight restoration	Arm, leg, trunk, android and gynoid fat mass percentages were lower than in controls, but no significant difference was found between the two groups in term of the android/gynoid ratio.	Preferential distribution of body fat in central regions (trunk, android), but such distribution did not appear to influence eating disorder psychopathology or psychological distress factors.
Mayo-Smith *et al.* [37]	1989	CT	Cross-sectional	15 AN females aged 15–33 years *vs.* 39 controls aged 18–35 years	-	Subcutaneous and visceral adipose tissue evaluated by CT. AN patients tend to lose more subcutaneous fat than intra-abdominal fat compared to controls	-
Zamboni *et al.* [38]	1997	CT	Longitudinal, 12-week inpatient treatment	21 adult AN females; no controls	Short-term partial weight restoration	Patients lost more subcutaneous adipose tissue than visceral adipose tissue	The increase in fat in subcutaneous abdominal tissue was significantly greater than visceral abdominal tissue.
Mayer *et al.* [39]	2009	MRI	Long-term longitudinal, 1-year follow-up after inpatient treatment	21 adult AN females *vs.* 10 age- and BMI-matched controls	Long-term weight maintenance	With acute weight restoration, AN patients had significantly greater visceral and intramuscular adipose tissue than control women	The abnormal fat distribution appeared to normalize within a 1-year period of weight maintenance
Orphanidou *et al.* [40]	1997	Skin-fold Thickness and DXA	Longitudinal, 20-week inpatient or 48-week outpatient	26 adult AN females *vs.* 21 controls	Short-term partial weight restoration	Not mentioned	BF distribution showed greater deposition in the central regions than in the extremities
Mayer *et al.* [14]	2005	AM DXA MRI	Longitudinal, inpatient treatment (4–17 weeks)	29 adult AN females *vs.* 15 age- and BMI-matched controls	Short-term complete weight restoration	At baseline, trunk/total fat was not statistically different from controls	Using different methods to assess body fat distribution suggested disproportionate central adipose tissue deposition

AN: Anorexia Nervosa; AN-R: restrictive Anorexia Nervosa; AM: anthropometric measurements; BF: body fat; CT: Computerized axial tomography; DXA: dual-energy X-ray absorptiometry; MRI: Magnetic resonance imaging.

## 3. Results

**Figure 1:**
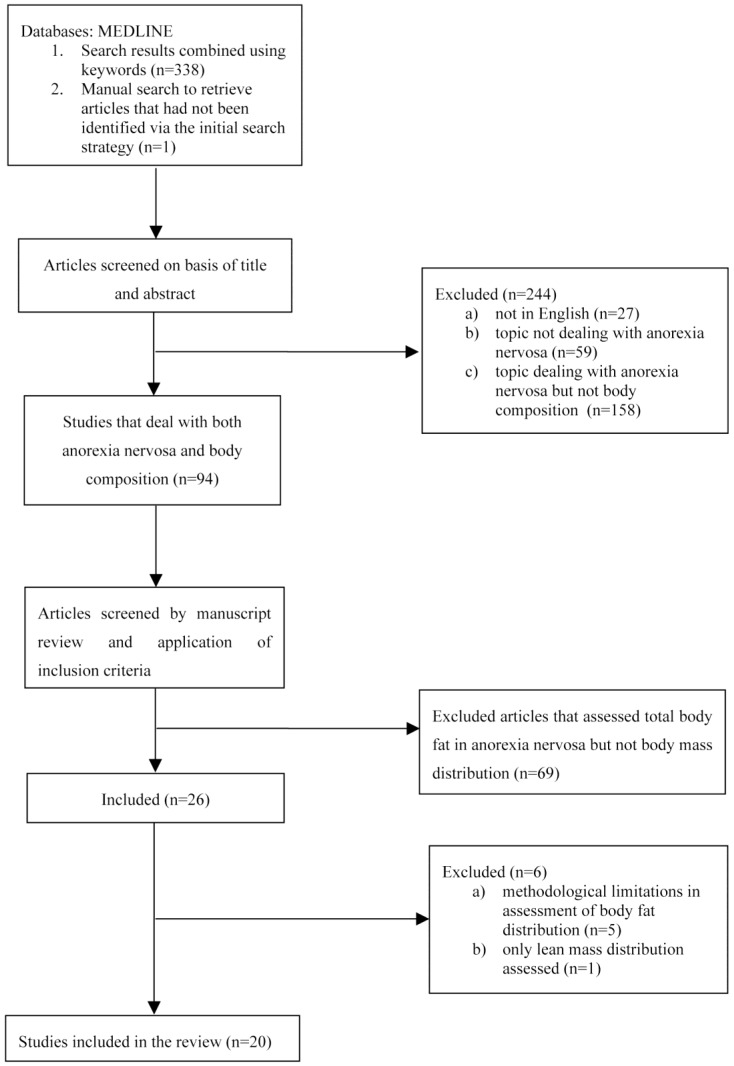
Flow chart summarizing the procedure for selecting studies.

The initial search retrieved 339 papers. After analyzing their titles and abstracts, 244 papers were excluded because: 27 were not in English; 59 were not concerned with anorexia nervosa; 158 dealt with anorexia nervosa but made no reference to or had no direct relationship with body composition assessment. In the second step, a further 69 papers were excluded because they investigated anorexia nervosa and body composition but not body mass distribution. Twenty-six articles remained, and were screened in light of their methodological quality and findings. At this stage, 6 studies were excluded for the following reasons: (I) methodological limitations in assessment of body fat distribution (*n* = 1); (II) small and heterogeneous sample including both adolescents and adults (age: 15–26 years) (*n* = 1); (III) assessment of endocrine changes rather than body fat distribution (*n* = 1); (IV) comparison of different devices to assess body fat in a heterogeneous group of females with eating disorders (*n* = 2); assessment of lean mass distribution rather than fat mass (*n* = 1) (Figure 1).

On the basis of this selection procedure, 20 articles (5 non-controlled longitudinal, 6 cross-sectional and 9 controlled longitudinal studies) were included in the systematic review and therefore underwent narrative analysis.

The NICE guidelines checklist proved a fair-to-good quality score for the non-controlled longitudinal and cross-sectional studies (*n* = 11) (mean score 5.36 points) (see Table S1 in online supporting material), while, judging by the Newcastle-Ottawa Scale checklist, the controlled longitudinal studies were of high quality (*n* = 9) (mean score 8.33 points) (see Table S2 in online supporting material).

### 3.1. Body Fat Distribution in Adolescents with Anorexia Nervosa

Forbes was the first to assess body fat distribution, in a cross-sectional study of 30 adolescent females with anorexia nervosa. Using the waist-to-hip ratio (WHR) as a measure of body fat distribution, a reduction in waist and hip circumferences was detected, but no difference in WHR was revealed between adolescents with anorexia nervosa and healthy controls [28].

A decade later, Kerruish *et al.* assessed body composition by means of dual-energy X-ray absorptiometry (DXA) in 23 adolescent females with anorexia nervosa, and reported a lower trunk, leg and trunk/leg fat ratio than 25 age-matched controls [22]. Misra *et al.* in addition, used DXA to measure regional body composition in two samples of 21 and 23 adolescent females with anorexia nervosa, finding a significantly lower percentage trunk fat (as a percentage of total fat) in adolescent females with anorexia nervosa than in healthy controls, whereas percentage extremity fat did not differ between the two groups [23,24]. More recently, De Alvaro *et al.* [25], also using DXA for assessment of body fat distribution, found a greater reduction of trunk and extremity fat masses in 42 adolescent females with restricting-type anorexia nervosa than in 23 healthy controls, but only the individuals affected by anorexia nervosa with prolonged malnutrition displayed a lower trunk/extremity fat ratio.

In contrast, Misra *et al.*, in the only DXA study to assess regional body composition in adolescent males with anorexia nervosa, found a higher percentage trunk fat and trunk/extremity fat ratio in their sample of 15 patients than in 15 controls, indicating a preferential fat loss in the extremities rather than the trunk (central, visceral fat), an effect attributed by the authors to the hypogonadal status and lower testosterone concentrations of males with anorexia nervosa [26].

As a whole, the data from these studies suggest that adolescent females with anorexia nervosa tend to lose more central/visceral fat (trunk) than peripheral fat (subcutaneous), while adolescent males seems to lose more peripheral fat.

### 3.2. Body Fat Distribution in Adults with Anorexia Nervosa

Mayo-Smith *et al.* used computerized axial tomography (CT) to measure subcutaneous and visceral abdominal fat in 15 females with anorexia nervosa, and 39 controls. Although limited by the cross-sectional nature of the study, the authors found that females with anorexia nervosa showed a five-fold reduction in subcutaneous abdominal fat but only a two-fold reduction in visceral abdominal fat with respect to controls [37]. Zamboni *et al.*, also using CT, extended Mayo-Smith’s findings in a non-controlled study of 21 patients, revealing that participants with anorexia nervosa had a greater absolute reduction in subcutaneous abdominal fat than visceral abdominal fat, specifically at the L4–L5 level [38]. 

Later on, Grinspoon *et al.* measured regional body fat, by means of DXA, in 27 women with anorexia nervosa with secondary amenorrhea, and in 20 age-matched controls. Percentage trunk fat did not differ significantly between the two groups, but women with anorexia nervosa had a significantly lower percentage extremity fat than controls [32]. In another DXA study, Mayer *et al.* assessed body fat distribution in 29 women with anorexia nervosa and 15 age- and BMI-matched controls, finding no significant differences in percentage trunk fat between the two groups [14]. This was confirmed by Prioletta *et al.*, who assessed regional body fat in 19 women with anorexia nervosa and 21 age-matched controls via DXA, and reported no difference in percentage trunk fat between the two groups [35]. Likewise, Grinspoon *et al.* found no difference in trunk/extremity fat ratio between underweight young adult females with anorexia nervosa and healthy controls [32]. Moreover, Kirchengast *et al.*, in two separate studies, used DXA to measure the Fat Distribution Index (*i.e.*, upper body fat mass/lower body fat mass) in 15 underweight adult females with anorexia nervosa and 19 age-matched healthy controls, reporting that although emaciated, the former group continued to show a gynoid fat pattern similar to the latter [29,33].

However, in our previous DXA investigation of body composition we found an increase in percentage trunk fat, together with a reduction in percentage arm fat, in 50 women with anorexia nervosa with respect to 100 healthy age and BMI-matched controls [36]. That being said, when we extended Kirchengast and Grinpsoon’s investigation, we confirmed that underweight adult females with anorexia nervosa had similar android/gynoid ratio (gynoid pattern) as healthy controls [36].

In summary, the data provided by the above studies indicate that adult females with anorexia nervosa tend to lose more peripheral (subcutaneous, extremity) than central fat (visceral, trunk, android), while apparently conserving a body fat distribution (waist to hip or android/gynoid ratios) similar to that of controls.

### 3.3. Body Fat Distribution after Partial and Complete Weight Restoration in Adolescents with Anorexia Nervosa

Only three DXA studies to date have assessed body fat distribution after partial [27] or complete weight restoration [23,25] in adolescent females with anorexia nervosa. The first, by Mirsa *et al.*, examined 13 subjects who achieved complete weight restoration (BMI >10th percentile) over 1 year, and found an increased percentage trunk fat but not percentage extremity fat. However, neither the percentage trunk fat nor the trunk/extremity fat ratio exceeded that of control subjects, indicating that the changes in adolescent girls with anorexia nervosa were likely to represent a normalization of body fat distribution [23]. The second study, by de Alvaro *et al.*, reported that complete weight restoration over 24 months with menses recovery in 15 adolescent females with restricting-type anorexia nervosa was associated with adequately distributed fat mass acquisition and no increase in central adiposity, and body fat distribution was similar to that of 23 controls [25]. The third study, by Franzoni *et al.*, assessed body fat patterns in 46 adolescent females with restricting-type anorexia nervosa after partial weight restoration, and found more evident deposition of fat in the trunk region (<0.001) than the arms (<0.05) and legs (=0.05), although no control group was available for comparison [27]. Nevertheless, long-term maintenance (12 and 24 months) of complete weight restoration does seem to be associated with adequately distributed fat mass acquisition and no increase in central adiposity, while partial weight restoration, as in adults, seems to be associated with a more evident deposition of fat in the trunk than in other regions.

### 3.4. Body Fat Distribution after Short-term Partial and Complete Weight Restoration in Adults with Anorexia Nervosa 

The distribution of body fat after short-term partial and complete weight restoration in adult females with anorexia nervosa has been widely studied. Zamboni *et al.* were the first to evaluate changes in regional body fat distribution in anorexia nervosa, assessing 21 adult females during 12 weeks of inpatient treatment and partial weight restoration. They focused specifically on the subcutaneous and visceral fat compartments of the abdominal region (L4–L5), and reported a 212% increase in subcutaneous fat and a 117% increase in visceral fat associated with a mean weight gain of 7.3 kg. Not surprisingly, they concluded that patients with anorexia nervosa gain abdominal fat during weight restoration, but were unable to discern preferential deposition of weight in the central regions because no other regions (*i.e.*, the extremities) were assessed. Another limitation of that study was that, even after an approximate weight gain of 7 kg, patients were still significantly underweight (BMI 17.5 + 2.0 kg/m^2^) at the time of reassessment [38].

In the same year, Orphanidou *et al.* assessed body fat distribution using skin-fold thickness and DXA in a group of 26 adult females with anorexia nervosa after short-term partial weight restoration, achieved through inpatient treatment (*n* = 21) or outpatient treatment (*n* = 5). Skin-fold thickness changes supported greater deposition of body fat in the central regions than in the extremities, but these anthropometric findings were not confirmed by DXA assessment changes in regional fat mass, something that still remains to be explained satisfactorily [40]. Iketani *et al.* extended the Orphanidou study by including a group of normal-weight control women in their DXA assessment of body fat distribution in 21 young women with anorexia nervosa. They found that short-term partial weight restoration brought about a trunk mass increase to levels similar to those of controls, but extremity fat remained significantly below control values [30].

Pagliato *et al.*, assessing changes in regional body fat in 17 young adult females with anorexia nervosa by means of DXA, reported that with a small weight gain (BMI from 13.2 ± 1.8 to 15.2 ± 1.9) there was a higher trunk fat deposition with respect to other body patterns within the patient group [31]. This ties in with DXA findings by Grinspoon *et al.*, who measured change in regional body fat longitudinally in a homogenous group composed of 20 young women with anorexia nervosa [32]. They reported that a small spontaneous weight gain (BMI from 16.1 ± 0.3 to 17.5 ± 0.4) was associated with a significant increase in percentage trunk fat without concomitant increase in extremity fat percentage. This correlated with urinary free cortisol (*r* = 0.66, *p* = 0.003) and occurred even though weight restoration was incomplete [32]. In addition, they found that in one subgroup (10 subjects) of their sample, oestrogen administration did not appear to alter such distribution [32]. As in previous studies, patients were still underweight at the end of this 9-month trial, making it impossible to evaluate body fat distribution upon complete weight restoration.

However, Mayer *et al.* attempted to replicate and extend these findings by studying the changes in regional body fat upon short-term complete weight restoration in 29 adult females with anorexia nervosa and 15 controls using anthropometric measures, DXA and MRI. They showed that short-term complete weight restoration led to disproportionately central adipose tissue deposition, elevated waist-to-hip ratio (*p* < 0.006), total trunk fat (*p* < 0.003), and visceral adipose tissue (*p* < 0.006) in patients with respect to controls [14]. More recently, Prioletta *et al.* used DXA to assess regional body fat after a small weight gain in 19 females with anorexia nervosa and 21 age-matched controls (BMI from 13.6 ± 1.8 to 14.4 ± 1.1). They also found preferential redistribution of fat mass in the trunk region with respect to controls [35]. Similarly, we used DXA to measure body composition, in a larger sample of 50 women with anorexia nervosa and 100 healthy age- and BMI-matched controls [36]. We showed that after short-term complete weight restoration, patients with anorexia nervosa had lower percentage arm fat, lower percentage leg fat, higher android/gynoid ratio and higher percentage trunk fat than controls.

In summary, the data from the above studies show that after short-term weight restoration, whether partial or complete, adult females with anorexia nervosa tend to accumulate trunk fat, which contributes to a predominantly central distribution of body fat.

### 3.5. Body Fat Distribution after Long-term Maintenance of Complete Weight Restoration in Anorexia Nervosa

Less is known about body fat distribution after long-term maintenance of complete weight restoration in anorexia nervosa patients. Mayer *et al.* used MRI to assess body fat distribution after one year of normal weight maintenance (BMI = 20.70 ± 2.32) in 16 adult females with anorexia nervosa, and in 10 healthy age- and BMI-matched controls. They found that the abnormal body fat distribution noticed after short-term complete weight restoration normalized within a one-year period of weight maintenance [39]. Similarly, Dellava *et al.* used DXA to assess body fat distribution in 16 recovered anorexia nervosa patients who had maintained normal weight (BMI = 21.9 ± 2.2) for 2 years or more [34]. They also found no difference in percentage trunk fat, percentage leg fat or percentage arm fat compared to 18 healthy age- and BMI-matched controls [34]. These two studies therefore suggest that long-term maintenance of completely restored weight is associated with a normalization of body fat distribution. 

### 3.6. Change in Body Fat Distribution, Metabolic Indices and Psychopathology 

Only two studies have thus far investigated the effect of changes in body fat distribution on metabolic indices and anorexia nervosa psychopathology. Prioletta *et al.* reported that the preferential truncal redistribution of fat mass with respect to controls was correlated with insulin resistance status, and strictly paralleled by a reduction in insulin sensitivity as compared to controls [35]. We, on the other hand, have shown that the preferential distribution of body fat in central regions associated with short-term complete weight restoration does not seem to significantly influence eating disorder psychopathology or psychological distress in patients with anorexia nervosa [36].

## 4. Discussion 

### 4.1. Summary of Evidence and Limitation 

The data of the studies included in this review can be summarized as following.

#### 4.1.1. Strong Evidence 

Strong evidence suggests that: (I) Adult females with anorexia nervosa lose more peripheral (subcutaneous, extremity) rather than central body fat (visceral, trunk, android) during the course of their illness [14,32,35,36]; (II) Adolescent females with anorexia nervosa, in contrast, lose more central (trunk, visceral) than peripheral body fat (subcutaneous, extremity) [22,23,24,25]; (III) Both adolescent and adult females, although emaciated, conserve a normal body fat distribution pattern (waist to hip or android/gynoid ratios) [28,29,32,33,36]; (IV) In adult females with anorexia nervosa, short-term partial and complete weight restoration is associated with preferential trunk fat accumulation with central adipose deposition [14,30,31,32,35,36,40]. None of these four findings require further replication.

#### 4.1.2. Evidence Still Requiring Confirmation 

Consistent evidence appears to suggest that: (I) The abnormal central adipose deposition appears to normalize after a long-term maintenance of complete weight restoration [34,39]; (II) After short-term complete weight restoration the preferential distribution of body fat in central regions does not seem to significantly influence eating disorder psychopathology or psychological distress in patients with anorexia nervosa [36]. These two findings need further confirmation through replicative studies, preferably with long-term follow up in large samples. 

#### 4.1.3. Weak Evidence

Weak evidence suggests that (I) Adolescent males with anorexia nervosa seem to display a preferential loss of fat from the extremities rather than the trunk (central, visceral), although data is derived from only one small cross-sectional study [26]; (II) Adolescent females with anorexia nervosa, like adult females, seem to have an adequately distributed fat mass acquisition and no increase of central adiposity after complete long-term maintenance of complete weight restoration, with similar body fat distribution to controls, but only two small studies of 13 and 15 participants, respectively, have yielded this finding [22,23]; (III) After partial weight restoration, adolescent females with anorexia nervosa show greater deposition of fat in the trunk than in other regions, although this data was provided by only one non-controlled study [27]. These reports require further investigation to clarify body fat distribution before and after long-term complete weight restoration in adolescent males and females.

#### 4.1.4. Paucity or Lack of Evidence

There is almost no data available on the metabolic consequences associated with the abnormal body fat distribution seen after partial or complete weight restoration in patients with anorexia nervosa. Only one study has reported that minimum short-term weight restoration showed a preferential redistribution of body fat in the trunk region with respect to controls, and that such distribution was associated with insulin resistance status [35]. No longer-term results are available on the development of metabolic and cardiovascular complications related to the increase in trunk fat, especially after complete weight restoration. However, in other populations (*i.e.*, the obese) an increase in central fat (*i.e.*, trunk or android) is associated with metabolic syndrome and an increased risk of mortality [41], as well as cardiovascular disease, non-alcoholic fatty liver disease, insulin resistance [42], infertility, and ovulation disorders [43]. In contrast, body fat deposition in gynoid area, particularly as it counters android fat deposition, is associated with enhanced metabolic health [15,44,45]. 

Another areas that lack evidence are the studies of body fat distribution in adolescent females with anorexia nervosa after short-term complete weight restoration, and in adult males with anorexia nervosa before and after weight restoration, which has not been yet investigated.

Finally, no study assessing the body fat distribution before and after weight loss in individuals with anorexia nervosa has yet been published. Indeed, since clinicians typically assess patients with anorexia nervosa when they are underweight, it is highly improbable that this study will be carried out in future. Unfortunately, the observation that visceral adipose tissue is lost preferentially with modest weight loss in overweight and obese individuals—an effect that is attenuated with greater weight loss [46]—cannot be translated to individuals with anorexia nervosa, whose weight loss tends to begin when they are normal weight.

### 4.2. Implications for Future Research 

Several areas for future research have been highlighted by this systematic review. The first is the physiological change in body fat distribution in individuals with anorexia nervosa. We still have very little data on body fat distribution in males with anorexia nervosa, and we urgently need more studies on the effect of short-term complete weight restoration in adolescents and long-term maintenance of complete weight restoration on body fat distribution in both adolescent and adult females with anorexia nervosa. We also need to conduct comparative studies between adolescent and adult females with anorexia nervosa using a uniform methodology (body composition instruments), where patients of both populations are undergoing the same treatment setting and duration, weight restoration rate, and nutritional and physical activity programs. The third area that requires investigation comprises the biochemical and biological mechanisms implicated in the changes of body fat distribution both before and after weight restoration. Finally, and in our opinion the most important from a clinical perspective, it that it is vital that we study the influence of fat distribution before and after partial and complete weight restoration on eating disorder psychopathology, treatment outcome, and metabolic and cardiovascular complications.

## 5. Conclusions

The information reported in this review has several clinical implications. First, during anorexia nervosa adolescent females with anorexia nervosa seem to lose more central body fat, while adult females more peripheral fat. Second, partial weight restoration leads to more fat mass deposition in the trunk region than other body regions in adolescents with anorexia nervosa. Third, after short-term weight restoration whether partial or complete, adult females show a central adiposity phenotype. Fourth, this abnormal fat distribution seen in adults with anorexia nervosa after weight restoration seems to be associated with an increase in insulin resistance, but it does not negatively influence eating disorder psychopathology, cause any psychological distress or affect treatment outcome. Fifth, body fat distribution seems to normalize after long-term maintenance of complete weight restoration. This finding, if confirmed, indicates that the preferential central distribution of body fat is a transitory phenomenon associated with the acute phase of weight regain.

Nevertheless readers will notice some discrepancies in the findings reported by the studies reviewed, especially between adolescents and adults. It is likely that these discrepancies are related to the following factors: (I) age differences—it is known that visceral fat is low in youth and tends to increase with age, especially in females [47]; (II) differences in the method of assessing body composition (e.g., conventional DXA cannot discriminate between visceral and subcutaneous trunk fat) [48]; (III) bias in body composition assessment timing after complete weight regain in adolescent females; (IV) and the heterogeneity of treatments used to achieve weight restoration.

## Supplementary Files

Supplementary File 1

## References

[1] Agras W.S. (2001). The consequences and costs of the eating disorders. Psychiatr. Clin. N. Am..

[2] Dalle Grave R. (2011). Eating disorders: Progress and challenges. Eur. J. Intern. Med..

[3] National Institute of Clinical Excellence (2004). Eating disorders. Core Interventions in the Treatment and Management of Anorexia Nervosa, Bulimia Nervosa and Related Eating Disorders.

[4] El Ghoch M., Alberti M., Milanese C., Battistini N.C., Pellegrini M., Capelli C., Calugi S., Dalle Grave R. (2012). Comparison between dual-energy X-ray absorptiometry and skinfolds thickness in assessing body fat in anorexia nervosa before and after weight restoration. Clin. Nutr..

[5] Krahn D.D., Rock C., Dechert R.E., Nairn K.K., Hasse S.A. (1993). Changes in resting energy expenditure and body composition in anorexia nervosa patients during refeeding. J. Am. Diet. Assoc..

[6] Probst M., Goris M., Vandereycken W., Van Coppenolle H. (1996). Body composition in female anorexia nervosa patients. Br. J. Nutr..

[7] American Psychiatric Association (2013). Diagnostic and Statistical Manual of Mental Disorders.

[8] Marzola E., Nasser J.A., Hashim S.A., Shih P.A., Kaye W.H. (2013). Nutritional rehabilitation in anorexia nervosa: Review of the literature and implications for treatment. BMC Psychiatry.

[9] Frisch R.E. (1988). Fatness and fertility. Sci. Am..

[10] Dalle Grave R., Pasqualoni E., Marchesini G. (2011). Symptoms of starvation in eating disorder patients. Handbook of Behavior, Food and Nutrition.

[11] Mayer L.E., Roberto C.A., Glasofer D.R., Etu S.F., Gallagher D., Wang J., Heymsfield S.B., Pierson R.N., Attia E., Devlin M.J. (2007). Does percent body fat predict outcome in anorexia nervosa?. Am. J. Psychiatry.

[12] Bodell L.P., Mayer L.E. (2011). Percent body fat is a risk factor for relapse in anorexia nervosa: A replication study. Int. J. Eat. Disord..

[13] Fairburn C.G. (2008). Cognitive Behavior Therapy and Eating Disorders.

[14] Mayer L., Walsh B.T., Pierson R.N., Heymsfield S.B., Gallagher D., Wang J., Parides M.K., Leibel R.L., Warren M.P., Killory E. (2005). Body fat redistribution after weight gain in women with anorexia nervosa. Am. J. Clin. Nutr..

[15] Folsom A.R., Kushi L.H., Anderson K.E., Mink P.J., Olson J.E., Hong C.P., Sellers T.A., Lazovich D., Prineas R.J. (2000). Associations of general and abdominal obesity with multiple health outcomes in older women: The Iowa Women’s Health Study. Arch. Intern. Med..

[16] Richardson W.S., Wilson M.C., Nishikawa J., Hayward R.S. (1995). The well-built clinical question: A key to evidence-based decisions. ACP J. Club..

[17] Liberati A., Altman D.G., Tetzlaff J., Mulrow C., Gotzsche P.C., Ioannidis J.P., Clarke M., Devereaux P.J., Kleijnen J., Moher D. (2009). The PRISMA statement for reporting systematic reviews and meta-analyses of studies that evaluate health care interventions: Explanation and elaboration. Ann. Intern. Med..

[18] (NICE) National Institute for Health and Clinical Excellence Clinical Guidelines, Appendix 4. Quality of Case Series Form. http://www.nice.org.uk/nicemedia/pdf/Appendix_04_qualityofcase_series_form_preop.pdf.

[19] Stang A. (2010). Critical evaluation of the Newcastle-Ottawa scale for the assessment of the quality of nonrandomized studies in meta-analyses. Eur. J. Epidemiol..

[20] Wang Y., Ji J., Liu Y.J., Deng X., He Q.Q. (2013). Passive smoking and risk of type 2 diabetes: A meta-analysis of prospective cohort studies. PLoS One.

[21] Popay J., Roberts H., Sowden A., Petticrew M., Britten N., Arai L., Roen K., Rodgers M. (2005). Developing guidance on the conduct of narrative synthesis in systematic reviews. J. Epidemiol. Community Health.

[22] Kerruish K.P., O’Connor J., Humphries I.R., Kohn M.R., Clarke S.D., Briody J.N., Thomson E.J., Wright K.A., Gaskin K.J., Baur L.A. (2002). Body composition in adolescents with anorexia nervosa. Am. J. Clin. Nutr..

[23] Misra M., Soyka L.A., Miller K.K., Grinspoon S., Levitsky L.L., Klibanski A. (2003). Regional body composition in adolescents with anorexia nervosa and changes with weight recovery. Am. J. Clin. Nutr..

[24] Misra M., Miller K.K., Almazan C., Worley M., Herzog D.B., Klibanski A. (2005). Hormonal determinants of regional body composition in adolescent girls with anorexia nervosa and controls. J. Clin. Endocrinol. Metab..

[25] De Alvaro M.T., Munoz-Calvo M.T., Barrios V., Martinez G., Martos-Moreno G.A., Hawkins F., Argente J. (2007). Regional fat distribution in adolescents with anorexia nervosa: Effect of duration of malnutrition and weight recovery. Eur. J. Endocrinol..

[26] Misra M., Katzman D.K., Cord J., Manning S.J., Mickley D., Herzog D.B., Miller K.K., Klibanski A. (2008). Percentage extremity fat, but not percentage trunk fat, is lower in adolescent boys with anorexia nervosa than in healthy adolescents. Am. J. Clin. Nutr..

[27] Franzoni E., Ciccarese F., Di Pietro E., Facchini G., Moscano F., Iero L., Monaldi A., Battista G., Bazzocchi A. (2014). Follow-up of bone mineral density and body composition in adolescents with restrictive anorexia nervosa: Role of dual-energy X-ray absorptiometry. Eur. J. Clin. Nutr..

[28] Forbes G.B. (1990). The abdomen: Hip ratio normative data and observations on selected patients. Int. J. Obes..

[29] Kirchengast S., Huber J. (1999). Body composition characteristics, sex hormone levels and circadian gonadotropin fluctuations in infertile young women. Coll. Antropol..

[30] Iketani T., Kiriike N., Nagata T., Yamagami S. (1999). Altered body fat distribution after recovery of weight in patients with anorexia nervosa. Int. J. Eat. Disord..

[31] Pagliato E., Corradi E., Gentile M.G., Testolin G. (2000). Changes in body composition and resting energy expenditure in anorectic patients after a weight gain of fifteen percent. Ann. N. Y. Acad. Sci..

[32] Grinspoon S., Thomas L., Miller K., Pitts S., Herzog D., Klibanski A. (2001). Changes in regional fat redistribution and the effects of estrogen during spontaneous weight gain in women with anorexia nervosa. Am. J. Clin. Nutr..

[33] Kirchengast S., Huber J. (2004). Body composition characteristics and fat distribution patterns in young infertile women. Fertil. Steril..

[34] Dellava J.E., Hamer R.M., Kanodia A., Reyes-Rodriguez M.L., Bulik C.M. (2011). Diet and physical activity in women recovered from anorexia nervosa: A pilot study. Int. J. Eat. Disord..

[35] Prioletta A., Muscogiuri G., Sorice G.P., Lassandro A.P., Mezza T., Policola C., Salomone E., Cipolla C., Della Casa S., Pontecorvi A. (2011). In anorexia nervosa, even a small increase in abdominal fat is responsible for the appearance of insulin resistance. Clin. Endocrinol. (Oxf.).

[36] El Ghoch M., Milanese C., Calugi S., Pellegrini M., Battistini N.C., Dalle Grave R. (2014). Body composition, eating disorder psychopathology, and psychological distress in anorexia nervosa: A longitudinal study. Am. J. Clin. Nutr..

[37] Mayo-Smith W., Hayes C.W., Biller B.M., Klibanski A., Rosenthal H., Rosenthal D.I. (1989). Body fat distribution measured with CT: Correlations in healthy subjects, patients with anorexia nervosa, and patients with Cushing syndrome. Radiology.

[38] Zamboni M., Armellini F., Turcato E., Todisco P., Gallagher D., Dalle Grave R., Heymsfield S., Bosello O. (1997). Body fat distribution before and after weight gain in anorexia nervosa. Int. J. Obes. Relat. Metab. Disord..

[39] Mayer L.E., Klein D.A., Black E., Attia E., Shen W., Mao X., Shungu D.C., Punyanita M., Gallagher D., Wang J. (2009). Adipose tissue distribution after weight restoration and weight maintenance in women with anorexia nervosa. Am. J. Clin. Nutr..

[40] Orphanidou C.I., McCargar L.J., Birmingham C.L., Belzberg A.S. (1997). Changes in body composition and fat distribution after short-term weight gain in patients with anorexia nervosa. Am. J. Clin. Nutr..

[41] Yusuf S., Hawken S., Ounpuu S., Bautista L., Franzosi M.G., Commerford P., Lang C.C., Rumboldt Z., Onen C.L., Lisheng L., Tanomsup S (2005). Obesity and the risk of myocardial infarction in 27,000 participants from 52 countries: A case-control study. Lancet.

[42] Carr M.C., Brunzell J.D. (2004). Abdominal obesity and dyslipidemia in the metabolic syndrome: Importance of type 2 diabetes and familial combined hyperlipidemia in coronary artery disease risk. J. Clin. Endocrinol. Metab..

[43] Diamanti-Kandarakis E., Bergiele A. (2001). The influence of obesity on hyperandrogenism and infertility in the female. Obes. Rev..

[44] Manolopoulos K.N., Karpe F., Frayn K.N. (2010). Gluteofemoral body fat as a determinant of metabolic health. Int. J. Obes. (Lond.).

[45] Okura T., Nakata Y., Yamabuki K., Tanaka K. (2004). Regional body composition changes exhibit opposing effects on coronary heart disease risk factors. Arterioscler. Thromb. Vasc. Biol..

[46] Chaston T.B., Dixon J.B. (2008). Factors associated with percent change in visceral versus subcutaneous abdominal fat during weight loss: Findings from a systematic review. Int. J. Obes. (Lond.).

[47] Zamboni M., Armellini F., Harris T., Turcato E., Micciolo R., Bergamo-Andreis I.A., Bosello O. (1997). Effects of age on body fat distribution and cardiovascular risk factors in women. Am. J. Clin. Nutr..

[48] Fosbøl M.Ø., Zerahn B. (2014). Contemporary methods of body composition measurement. Clin. Physiol. Funct. Imaging.

