# Supplementary material for: Anorexia Nervosa and Body Fat Distribution: A Systematic Review
Source: Nutrients. 2014 Sep 23;6(9):3895–912. doi: 10.3390/nu6093895 (PMC4179194; doi:10.3390/nu6093895)
Supplement: Supplementary File 1 [file nutrients-06-03895-s001.docx]

**Supplementary Materials**

**Table S1.** Quality assessment of non-controlled longitudinal studies and cross-sectional studies.

| **Author** | **Mayo-Smith ****  **1989** | **Forbes ***  **1990** | **Orphanidou** **1997** | **Zamboni ***  **1997** | **Kirchengast **** **1999** | **Pagliato ***  **2000** | **Kerruish **** **2002** | **Kirchengast **** **2003** | **Misra ****  **2008** | **Dellava ****  **2010** | **Franzoni ***  **2014** |
| --- | --- | --- | --- | --- | --- | --- | --- | --- | --- | --- | --- |
| Case series collected in more than one center, *i.e.*, multi-center study | 0 | 0 | 0 | 0 | 0 | 0 | 1 | 0 | 1 | 0 | 0 |
| Is the hypothesis/aim/objective of the study clearly described? | 1 | 1 | 1 | 1 | 1 | 1 | 1 | 1 | 1 | 1 | 1 |
| Are the inclusion and exclusion criteria (case definition) clearly reported? | 0 | 1 | 0 | 0 | 1 | 0 | 1 | 1 | 1 | 1 | 1 |
| Is there a clear definition of the outcomes reported? | 1 | 1 | 1 | 1 | 1 | 1 | 1 | 1 | 1 | 1 | 1 |
| Were data collected prospectively? | 1 | 1 | 1 | 1 | 0 | 0 | 1 | 0 | 1 | 1 | 1 |
| Is there an explicit statement that patients were recruited consecutively? | 0 | 0 | 0 | 0 | 0 | 0 | 0 | 0 | 0 | 0 | 0 |
| Are the main findings of the study clearly described? | 1 | 1 | 1 | 1 | 1 | 1 | 1 | 1 | 1 | 1 | 0 |
| Are outcomes stratified? (e.g., by disease stage, abnormal test results,  patient characteristics) | 1 | 1 | 1 | 1 | 1 | 1 | 1 | 1 | 1 | 1 | 0 |
| **Total Score** | **5** | **6** | **5** | **5** | **5** | **4** | **7** | **5** | **7** | **6** | **4** |

NICE guidelines checklist: Yes = 1, No (not reported, not available) = 0; Total score, 8; ≤3, poor quality; 4–6, fair quality; ≥7, good quality; * Non-controlled longitudinal study;
** Cross-sectional study.

**Table S2.** Quality assessment of controlled longitudinal studies.

| **Author** | **Iketani**  **1999** | **Grinspoon**  **2000** | **Misra**  **2003** | **Mayer**  **2005** | **Misra**  **2005** | **De** **Alvaro**  **2007** | **Mayer**  **2009** | **Prioletta**  **2011** | **El** **Ghoch**  **2014** |
| --- | --- | --- | --- | --- | --- | --- | --- | --- | --- |
| **Selection** | | | | | | | | | |
| Represents cases with independent validation | 1 | 1 | 1 | 1 | 1 | 1 | 1 | 1 | 1 |
| Cases are consecutive or obviously representative | 1 | 1 | 0 | 1 | 1 | 1 | 1 | 1 | 1 |
| Controls are from community | 1 | 1 | 1 | 1 | 1 | 1 | 1 | 1 | 1 |
| Controls have no history of Anorexia Nervosa | 1 | 1 | 1 | 1 | 1 | 1 | 1 | 1 | 1 |
| **Comparability** | | | | | | | | | |
| Controls are comparable for the most important factors | 1 | 1 | 1 | 1 | 1 | 1 | 1 | 1 | 1 |
| Control for any additional factor | 0 | 0 | 1 | 1 | 1 | 0 | 1 | 0 | 1 |
| **Ascertainment of Exposure** | | | | | | | | | |
| Secured record or structured interview where blind to case/control status | 1 | 1 | 1 | 1 | 1 | 1 | 1 | 1 | 1 |
| Same method of ascertainment for cases and controls | 1 | 1 | 1 | 1 | 1 | 1 | 1 | 1 | 1 |
| Cases and controls have completed follow up | 1 | 1 | 1 | 1 | 1 | 1 | 1 | 0 | 1 |
| **Total Score** | **8** | **8** | **8** | **9** | **9** | **8** | **9** | **7** | **9** |

Newcastle-Ottawa Scale (NOS) for longitudinal case control studies. Yes = 1, No (not reported, not available) = 0; Studies with scores of 0–3, 4–6, 7–9 were considered as low, moderate and high quality, respectively.

© 2014 by the authors; licensee MDPI, Basel, Switzerland. This article is an open access article distributed under the terms and conditions of the Creative Commons Attribution license (http://creativecommons.org/licenses/by/3.0/).
